# Bisbenzamidines as Antifungal Agents. Are Both Amidine Functions Required to Observe an Anti-*Pneumocystis carinii* Activity?

**DOI:** 10.3390/molecules15064283

**Published:** 2010-06-11

**Authors:** Julien Laurent, Dimitri Stanicki, Tien L. Huang, Eduardo Dei-Cas, Muriel Pottier, El Mouktar Aliouat, Jean Jacques Vanden Eynde

**Affiliations:** 1 Laboratory of Organic Chemistry, Faculty of Sciences, University of Mons-UMONS, 20 place du parc, B-7000 Mons, Belgium; 2 Division of Basic Pharmaceutical Sciences, College of Pharmacy, Xavier University of Louisiana, 1 Drexel drive, New Orleans, LA 70125, USA; 3 Department of Parasitology-Mycology, Faculty of Biological and Pharmaceutical Sciences, University of Lille Nord de France, Lille, France; 4 Department of Parasitology-Mycology, Faculty of Medicine, University of Lille Nord de France, Biology-Pathology Centre, University Hospital Center, Lille, France; 5 Biology and Diversity of Emergent Eukaryotic Pathogens (BDEEP) (EA3609), IFR142, Institut Pasteur de Lille, Lille, France

**Keywords:** pentamidine, *Pneumocystis carinii*, monobenzamidines

## Abstract

A library of 19 novel 4-(4-phenylpiperazine-1-yl)benzamidines has been synthesized and evaluated *in vitro* against *Pneumocystis carinii*. Among these compounds, *N*-ethyl- and *N*-hexyl-4-(4-phenylpiperazine-1-yl)benzamidines emerged as the most promising compounds, with inhibition percentages at 10.0 µg/mL of 87% and 96%, respectively. Those compounds remained active at 0.1 µg/mL.

## 1. Introduction 

**Figure 1:**
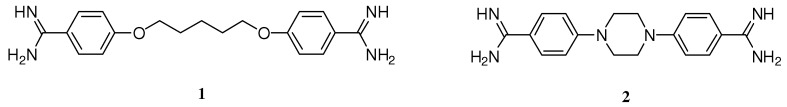
Structure of pentamidine (**1**) and piperazine-1, 4-bisbenzamidine (**2**).

*Pneumocystis jirovecii* pneumonia is a fungal disease that affects immunodeficient individuals and remains an important cause of mortality in AIDS infected persons [1]. The parasite does not respond to classical antifungal therapy, but it is sensitive to some antiprotozoal medicines [2]. Currently, available anti-*Pneumocystis* drugs are limited by significant problems of efficacy, toxicity and emerging resistance. Classical treatments to cure the disease include the well-known trimethoprim-sulfamethoxazole association (TMP-SMX; Bactrim^TM^, Septra^®^), dapsone (Avlosulfon^®^), atovaquone (Mepron^®^), and pentamidine (NebuPent^®^, Pentacarinat^®^) (**1**, Figure 1). Among those medicines, pentamidine remains the most effective drug to cure *Pneumocystis* pneumonia. However major side effects [1] and a poor bioavailability limit its use.

From a structural point of view, pentamidine (**1**, Figure 1) can be considered as a bisbenzamidine derivative in which both benzamidine moieties are linked by a highly flexible pentyldioxy chain. The structural simplicity of the molecule and its efficacy have encouraged some laboratories [3,4,5,6] to prepare original analogues with the hope to design more efficient and less toxic agents. In previous work [7,8] we demonstrated that 4, 4’-(1,4-piperazinediyl)bisbenzenecarboximidamide (**2,** Figure 1) is a promising candidate characterized by an *in vitro* IC_50_ of 2.61 µM against *P. carinii* (the rat-associated specie) and exhibiting no *in vitro* cytotoxicity. In addition, we observed that the introduction of alkyl substituents on the nitrogen atoms of the amidine functions of **2** could increase up to 1,000 fold the activity of the parent compound [7,8].

The mechanism of action of pentamidine and other bisbenzamidines analogues remains unclear. Because bisbenzamidines were shown to bind to the minor groove of DNA [9,10,11], it was initially thought that the anti-*Pneumocystis* properties were linked to the parasite replication. However derivatives exhibiting high anti-*P. carinii* activity and a poor affinity for DNA binding have already been identified. A mitochondrial toxicity has been postulated since it has been shown that same bisbenzamidines, including pentamidine, could form complexes with heme [12].

**Figure 2:**
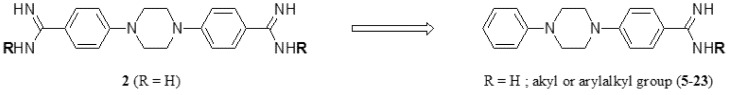
General structure of the monobenzamidines **5**-**23** prepared in this study.

In order to gain insight into Structure Activity Relationships (SAR) of bisbenzamidines, we decided to prepare a library of monobenzamidines structurally related to compound **2** (Figure 2) and to evaluate their biological behavior against *P. carinii*.

## 2. Results and Discussion 

### 2.1. Chemistry

**Scheme 1:**
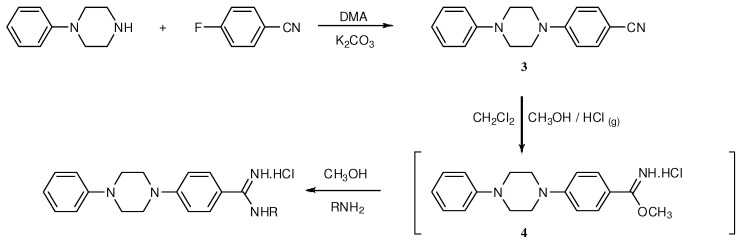
Synthesis of compounds **3**-**23**.

The first step in the preparation of such compounds is the nucleophilic displacement of the fluorine atom in 4-fluorobenzonitrile by the secondary amine function of 4-phenylpiperazine in refluxing DMA in the presence of K_2_CO_3_ as a base (Scheme 1). This step could advantageously be performed in a laboratory microwave oven so that reaction time can be reduced from 5 hours to 60 minutes.

Conversion of the nitrile compound **3** into the amidines **5**–**23** was effected by the Pinner reaction [13], whereby a solution of compound **3** in dichloromethane saturated with gaseous hydrochloric acid was treated with methanol to afford the imidate intermediate (Scheme 1). This compound was finally reacted with the appropriate amine to give analytically pure amidines. 

### 2.2. Biological evaluation

Table 1 contains the results of the *in vitro* evaluation of pentamidine **1**, compound **2**, and the benzamidines **5**-**23** against *P. carinii*. Analysis of the data indicated that all compounds retain an antifungal activity at a concentration of 10 µg/mL, with inhibition percentage ranging from 56% to 96%.

At a low concentration of 0.1 µg/mL, the results are more contrasted. At that concentration, the data suggest that the activity was dramatically dependent on the nature of the substituent on the amidine function, as previously described in the bisbenzamidine series [3,4]. In particular, we observed that the presence of an arylalkyl substituent on the amidine function (compounds **19**-**23**) led to a loss of the antifungal activity. Among the other derivatives, the most active compounds were those bearing a linear alkyl group of 1, 2, 3, or 6 carbon atoms (compounds **6**-**8**; **15**). Whereas piperazine-1,4-bisbenzamidine (compound **2**) and most of its *N*-alkyl substituted congeners previously studied were at least as efficient as pentamidine (compound **1**), the situation is a little bit different in the monobenzamidine series described in the present work. Indeed, all derivatives are less active than pentamidine. Starting from the unsubstituted compound, introduction of an alkyl chain of increasing length on the amidine function leads to a modulation of the anti-*Pneumocystis* activity, with a maximum of activity observed in the case of ethyl and hexyl substituents. Interestingly, compounds bearing an alkyl chain constituted by 7, 8, or 12 carbon atoms are not active against the fungus, contrary to the bisbenzamidine series. Mention should also be made that in the bisbenzamidine series as well as in the monobenzamidine series, the *N*-hexyl substituted candidates emerged among the most promising substances. That can tentatively be attributed to a favorable compromise between the hydrophilic properties of the amidine function(s) and the lipophilic character of the alkyl chain(s). 

Contrary to the observation made in the bisbenzamidine series, the substitution of the amidines group by an alkyl ramified chain led to a loss of the antifungal activity, except for the analogue bearing a 1-ethylpropyl substituent.

## 3. Experimental

### 3.1. General

^1^H-NMR spectra were obtained using a Bruker AMX instrument (300 MHz), chemical shifts (δ) are given in ppm using TMS as internal reference. The following abbreviations are used: br for broad, s for singlet, d for doublet, t for triplet, q for quadruplet, and m for multiplet. IR spectra were recorded on a Perkin-Elmer FTIR 1760K. Microwave synthesis were performed in a Milestone Multisynth® oven. Solvents, reagents, and pentamidine (**1**) were commercially available (Aldrich, Alfa Aesar, Acros Organics) and were used without further purification. Compounds **2** [14] and **3** [15] have been described in the literature. Elemental analyses were performed at the Centre Wallon de Recherches Agronomiques (Libramont-Chevigny, Belgium) or at the Laboratoire de Microanalyse Organique of the Institut des Sciences Appliqués de Rouen (France).

### 3.2. General procedure for the preparation of compound 3 under microwave irradiation

A mixture of 4-fluorobenzonitrile (2.5 mmol; 0.30 g) and 1-phenylpiperazine (2.5 mmol; 0.38 mL; 0.41 gr) in DMA (2.50 mL) in the presence of K_2_CO_3_ (2.5 mmol; 0.35 g) was heated 60 minutes at 140 °C in a Multisynth® oven (Milestone) operating at 300 watts. After cooling, the solution was poured into ice cold water and the precipitate was filtered and washed with water and ethanol.

### 3.3. General procedure for the preparation of compounds ***4**–**23***

A mixture of 4-(4-phenylpiperazine-1-yl)benzonitrile (**3**, 10 mmol, 2.66 g) in dichloromethane (250 mL) and methanol (25 mL) was saturated with HCl gas and the reaction medium was left at room temperature for 24 hours. The precipitate was filtered and thoroughly washed with ether. Without further purification the crude imidate **4** (3 mmol, 1.0 g) was treated with the appropriate amine in refluxing methanol for 1 hour. A precipitate was obtained either by cooling or by addition of ether. 

*4-(4-Phenylpiperazine-1-yl)benzenecarboximidamide hydrochloride salt* (**5**). Prepared by treatment of the crude imidate **4** with ammonia (15 mmol, 2.5 mL of a 7 N methanolic solution) in refluxing ethanol (10 mL) for 1 hour. The precipitate obtained after cooling was filtered and washed with ether and water. Yield: 64%. M.p.: >300 °C; ^1^H-NMR (DMSO-*d_6_*): 9.2 (br, 2H), 9.0 (br, 2H), 7.8 (d, 2H, *J* = 9 Hz), 7.3 (t, 2H, *J* = 7 Hz), 7.1 (d, 2H, *J* = 9 Hz), 7.0 (d, 2H, *J* = 7 Hz), 6.8 (t, 1H, *J* = 7 Hz), 3.5 (t, 4H, *J* = 5 Hz), 3.3 (t, 4H, *J* = 5 Hz) ppm; IR: 3,073, 2,834, 1,658, 1,607, 1,698, 1,494 cm^-1^; C_17_H_20_N_4_·HCl (316.15). Calc.: C, 63.68; H, 6.63; N, 17.47. Found: C, 64.03; H, 6.23; N, 17.37.

*N-Methyl 4-(4-phenylpiperazine-1-yl)benzenecarboximidamide hydrochloride salt* (**6**). Prepared by treatment of the crude compound **4** with methylamine (15 mmol, 1.8 mL of an ethanolic solution at 33%) in ethanol (10 mL) at reflux for 1 hour. After cooling, the precipitate obtained by addition of ether was filtered and washed with water. Yield: 57%. M.p.: >300 °C; ^1^H-NMR (DMSO-*d_6_*): 9.7 (br, 1H), 9.3 (br, 1H), 9.0 (br, 1H), 7.8 (d, 2H, *J* = 9 Hz), 7.3 (t, 2H, *J* = 7 Hz), 7.1 (d, 2H, *J* = 9 Hz), 7.0 (d, 2H, *J* = 7 Hz), 6.8 (t, 1H, *J* = 7 Hz), 3.5 (t, 4H, *J* = 5 Hz), 3.3 (t, 4H, *J* = 5 Hz), 3.0 (s, 3H) ppm; IR: 3,441, 3,056, 1,666, 1,505, 1,446, 1,367, 1,236 cm^-1^; C_18_H_22_N_4_·HCl (330.16). Calc.: C, 65.34; H, 7.01; N, 16.93. Found: C, 65.52; H, 6.99; N, 16.96.

*N-Ethyl 4-(4-phenylpiperazine-1-yl)benzenecarboximidamide hydrochloride salt* (**7**). Prepared by treatment of the crude imidate **4** with ethylamine (15 mmol, 7.5 mL of a 2 M methanolic solution) in refluxing methanol (10 mL) for 1 hour. After cooling, the precipitate obtained by addition of ether was filtered and washed with water. Yield: 47%. M.p.: 280–284 °C; ^1^H- NMR (DMSO-*d_6_*): 8.8 (br, 3H), 7.8 (d, 2H, *J* = 9 Hz), 7.3 (t, 2H, *J* = 7 Hz), 7.1 (d, 2H, *J* = 9 Hz), 7.0 (d, 2H, *J* = 7 Hz), 6.8 (t, 1H, *J* = 7 Hz), 3.5 (t, 4H, *J* = 5 Hz), 3.4 (q, 2H, *J* = 7 Hz), 3.3 (t, 4H, *J* = 5 Hz), 1.2 (t, *J* = 7 Hz, 3H) ppm; IR: 3,051, 2,840, 1,672, 1,604, 1,505, 1,387, 1,235 cm^-1^; C_19_H_24_N_4_·HCl (344.18). Calc.: C, 66.17; H, 7.31; N, 16.25. Found: C, 65.87; H, 7.27; N, 15.99.

*N-Propyl 4-(4-phenylpiperazine-1-yl)benzenecarboximidamide hydrochloride salt* (**8**). Prepared by treatment of the crude imidate **4** with propylamine (15 mmol, 1.2 mL) in refluxing ethanol (10 mL) for 1 hour. The precipitate obtained after cooling was filtered and washed with ether and water. Yield: 53%. M.p.: 275–280 °C; ^1^H-NMR (DMSO-*d_6_*): 9.3 (br, 3H), 7.8 (d, 2H, *J* = 9 Hz), 7.3 (t, 2H, *J* = 7 Hz), 7.1 (d, 2H, *J* = 9 Hz), 7.0 (d, 2H, *J* = 7 Hz), 6.8 (t, 1H, *J* = 7 Hz), 3.5 (t, 4H, *J* = 5 Hz), 3.4 (q, 2H, *J* = 7 Hz), 3.3 (t, 4H, *J* = 5 Hz), 1.7 (m, 2H, *J* = 7 Hz), 0.9 (t, 3H, J = *7* Hz); IR: 3,052, 2,970, 2,872, 1,670, 1,505, 1,452, 1,361, 1,234 cm^-1^; C_20_H_26_N_4_·HCl (358.19). Calc.: C, 66.93; H, 7.58; N, 15.61. Found: C, 66.99; H, 7.33; N, 15.39.

*N-Isopropyl 4-(4-phenylpiperazine-1-yl)benzenecarboximidamide hydrochloride salt* (**9**). Prepared by treatment of the crude imidate **4** with isopropylamine (15 mmol, 1.3 mL) in refluxing ethanol (10 mL) for 1 hour. After cooling, the precipitate obtained by addition of ether was filtered and washed with water. Yield: 67%. M.p.: >300 °C; ^1^H-NMR (DMSO-*d_6_*): 8.7 (br, 3H), 7.8 (d, 2H, *J* = 9 Hz), 7.3 (t, 2H, *J* = 7 Hz), 7.1 (d, 2H, *J* = 9 Hz), 7.0 (d, 2H, *J* = 7 Hz), 6.8 (t, 1H, *J* = 7 Hz), 4.1 (m, 1H, *J* = 7 Hz), 3.5 (t, 4H, *J* = 5 Hz), 3.3 (t, 4H, *J* = 5 Hz), 1.3 (d, 6H, *J* = 5 Hz); IR: 3,410, 3,051, 2,971, 1,666, 1,601, 1,505, 1,385, 1,234 cm^-1^; C_20_H_26_N_4_·HCl (358.19). Calc.: C, 66.93; H, 7.58; N, 15.61. Found: C, 67.02; H, 7.63; N, 15.57.

*N-Butyl 4-(4-phenylpiperazine-1-yl)benzenecarboximidamide hydrochloride salt* (**10**). Prepared by treatment of the crude imidate **4** with butylamine (15 mmol, 1.3 mL) in refluxing ethanol (10 mL) for 1 hour. After cooling, the precipitate obtained by addition of ether was filtered and washed with water. Yield: 32%. M.p.: 280–284 °C; ^1^H-NMR (DMSO-*d_6_*): 9.3 (br, 3H), 7.8 (d, 2H, *J* = 9 Hz), 7.3 (t, 2H, *J* = 7 Hz), 7.1 (d, 2H, *J* = 9 Hz), 7.0 (d, 2H, *J* = 7 Hz), 6.8 (t, 1H, *J* = 7 Hz), 3.5 (t, 4H, *J* = 5 Hz), 3.4 (t, 2H, *J* = 7 Hz), 3.3 (t, 4H, *J* = 5 Hz), 1.6 (m, 2H, *J* = 7 Hz), 1.4 (m, 2H, *J* = 7 Hz), 0.9 (t, 3H, *J* = 7 Hz); IR: 3,229, 3,091, 2,951, 1,665, 1,614, 1,520, 1,497, 1,386, 1,225 cm^-1^; C_21_H_28_N_4_·HCl (372.21). Calc.: C, 67.63; H, 7.84; N, 15.02. Found: C, 67.59; H, 7.85; N, 15.03.

*N-Pentyl 4-(4-phenylpiperazine-1-yl)benzenecarboximidamide hydrochloride salt* (**11**). Prepared by treatment of the crude imidate **4** with pentylamine (15 mmol, 1.7 mL) in refluxing ethanol (10 mL) for 1 hour. After cooling, the precipitate obtained by addition of ether was filtered and washed with water. Yield: 30%. M.p.: 285–290 °C. ^1^H NMR (DMSO-*d_6_*): 9.3 (br, 3H), 7.7 (d, 2H, *J* = 9 Hz), 7.3 (t, 2H, *J* = 7 Hz), 7.1 (d, 2H, *J* = 9 Hz), 7.0 (d, 2H, *J* = 7 Hz), 6.8 (t, 1H, *J* = 7 Hz), 3.5 (t, 4H, *J* = 5 Hz), 3.4 (t, 2H, *J* = 7 Hz), 3.3 (t, 4H, *J* = 5 Hz), 1.6 (m, 2H, *J* = 7 Hz), 1.3 (m, 4H), 0.9 (t, 3H, *J* = 7 Hz). IR: 3063, 2957, 2857, 1662, 1603, 1505, 1451, 1386, 1336, 1231 cm^-1^. C_22_H_30_N_4_·1.2 HCl (386,22). Calc.: C, 67.02; H, 7.98; N, 14.21. Found: C, 66.88; H, 8.04; N, 14.45.

*N-(3-Methylbutyl)l 4-(4-phenylpiperazine-1-yl)benzenecarboximidamide hydrochloride salt* (**12**). Prepared by treatment of the crude imidate with 3-methylbutylamine (15 mmol, 1.7 mL) in refluxing *ethanol* (10 mL) for 1 hour. After cooling, the precipitate obtained by addition of ether was filtered and washed with water. Yield: 63%. M.p.: >300 °C; ^1^H-NMR (DMSO-*d_6_*): 9.3 (br, 3H), 7.8 (d, 2H, *J* = 9 Hz), 7.3 (t, 2H, *J* = 7 Hz), 7.1 (d, 2H, *J* = 9 Hz), 7.0 (d, 2H, *J* = 7 Hz), 6.8 (t, 1H, *J* = 7 Hz), 3.5 (t, 4H, *J* = 5 Hz), 3.4 (t, 2H, *J* = 7 Hz), 3.3 (t, 4H, *J* = 5 Hz), 1.7 (m, 1H), 1.6 (m, 2H), 0.9 (d, 6H, J = 7 Hz); IR: 3,063, 2,957, 2,857, 1,662, 1,603, 1,505, 1,451, 1,386, 1,336, 1,231 cm^-1^; C_22_H_30_N_4_·HCl (386.22). Calc.: C, 68.28; H, 8.07; N, 14.48. Found: C, 68.08; H, 7.84; N, 14.22.

*N-(2-Methylbutyl)l 4-(4-phenylpiperazine-1-yl)benzenecarboximidamide hydrochloride salt* (**13**). Prepared by treatment of the crude imidate **4** with 2-methylbutylamine (15 mmol, 1.8 mL) in refluxing ethanol (10 mL) for 1 hour. The precipitate obtained after cooling was filtered and washed with ether and water. Yield: 35%. M.p.: >300 °C; ^1^H-NMR (DMSO-*d_6_*): 9.3 (br, 3H), 7.8 (d, 2H, *J* = 9 Hz), 7.3 (t, 2H, *J* = 7 Hz), 7.1 (d, 2H, *J* = 9 Hz), 7.0 (d, 2H, *J* = 7 Hz), 6.8 (t, 1H, *J* = 7 Hz), 3.5 (t, 4H, *J* = 5 Hz), 3.4 (d, 2H, *J* = 7 Hz), 3.3 (t, 4H, *J* = 5 Hz), 1.8 (m, 1H), 1.2 (m, 2H), 0.9 (t, 3H, *J* = 7 Hz), 0.9 (d, 3H, *J* = 6 Hz); IR: 3,024, 2,960, 2,844, 1,671, 1,604, 1,515, 1,450, 1,387, 1,230 cm^-1^; C_22_H_30_N_4_·1.1 HCl (386.22). Calc.: C, 67.65; H, 8.03; N, 14.34. Found: C, 67.44; H, 7.80; N, 14.11.

*N-(1-Ethylpropyl) 4-(4-phenylpiperazine-1-yl)benzenecarboximidamide hydrochloride salt* (**14**). Prepared by treatment of the crude imidate **4** with 1-ethylpropylamine (15 mmol, 1.8 mL) in refluxing ethanol (10 mL) for 1 hour. The precipitate obtained after cooling was filtered and washed with ether and water. Yield: 61%. M.p.: >300 °C; ^1^H-NMR (DMSO-*d_6_*): 9.3 (br, 3H), 7.8 (d, 2H, *J* = 9 Hz), 7.3 (t, 2H, *J* = 7 Hz), 7.1 (d, 2H, *J* = 9 Hz), 7.0 (d, 2H, *J* = 7 Hz), 6.8 (t, 1H, *J* = 7 Hz), 3.8 (m, 1H), 3.5 (t, 4H, *J* = 5 Hz), 3.3 (t, 4H, *J* = 5 Hz), 1.6 (m, 4H), 0.9 (t, 6H, *J* = 7 Hz); IR: 3,074, 2,966, 2,935, 2,880, 1,666, 1,600, 1,505, 1,231 cm^-1^; C_22_H_30_N_4_·HCl (386.22). Calc.: C, 68.28; H, 8.07; N, 14.48. Found: C, 68.15; H, 8.08; N, 14.46.

*N-Hexyl 4-(4-phenylpiperazine-1-yl)benzenecarboximidamide hydrochloride salt* (**15**). The compound was prepared by treatment of the crude imidate **4** with hexylamine (15 mmol, 2.0 mL) in refluxing ethanol (10 mL) for 1 hour. The precipitate obtained after cooling was filtered and washed with ether and water. Yield: 42%. M.p.: 285–290 °C; ^1^H-NMR (DMSO-*d_6_*): 9.3 (br, 3H), 7.7 (d, 2H, *J* = 9 Hz), 7.3 (t, 2H, *J* = 7 Hz), 7.1 (d, 2H, *J* = 9 Hz), 7.0 (d, 2H, *J* = 7 Hz), 6.8 (t, 1H, *J* = 7 Hz), 3.5 (t, 4H, *J* = 5 Hz), 3.4 (t, 2H, *J* = 7 Hz), 3.3 (t, 4H, *J* = 5 Hz), 1.6 (m, 2H, *J* = 7 Hz), 1.3 (m, 6H), 0.9 (t, 3H, *J* = 7 Hz); IR: 3,050, 2,956, 2,931, 1,669, 1,505, 1,451, 1,387, 1,232 cm^-1^; C_23_H_32_N_4_·1.1 HCl (400.24). Calc.: C, 68.27; H, 8.25; N, 13.85. Found: C, 68.31; H, 8.19; N, 13.98.

*N-Heptyl 4-(4-phenylpiperazine-1-yl)benzenecarboximidamide hydrochloride salt* (**16**). Prepared by treatment of the crude imidate **4** with heptylamine (15 mmol, 2.2 mL) in refluxing ethanol (10 mL) for 1 hour. After cooling, the precipitate obtained by addition of ether was filtered and washed with water. Yield: 43%. M.p.: 290–294 °C; ^1^H-NMR (DMSO-*d_6_*): 9.3 (br, 3H), 7.7 (d, 2H, *J* = 9 Hz), 7.3 (t, 2H, *J* = 7 Hz), 7.1 (d, 2H, *J* = 9 Hz), 7.0 (d, 2H, *J* = 7 Hz), 6.8 (t, 1H, *J* = 7 Hz), 3.5 (t, 4H, *J* = 5 Hz), 3.4 (t, 2H, *J* = 7 Hz), 3.3 (t, 4H, *J* = 5 Hz), 1.6 (m, 2H, *J* = 7 Hz), 1.3 (m, 8H), 0.9 (t, 3H, *J* = 7 Hz); IR: 3,078, 2,956, 2,926, 1,661, 1,505, 1,387, 1,387, 1,234 cm^-1^; C_24_H_34_N_4_·1.1 HCl (414.26). Calc.: C, 68.85; H, 8.45; N, 13.38. Found: C, 69.20; H, 8.09; N, 13.47.

*N-Octyl 4-(4-phenylpiperazine-1-yl)benzenecarboximidamide hydrochloride salt* (**17**). Prepared by treatment of the crude imidate **4** with octylamine (15 mmol, 2.5 mL) in refluxing ethanol (10 mL) for 1 hour. The precipitate obtained after cooling was filtered and washed with ether and water. Yield: 43%. M.p.: 280–284 °C; ^1^H-NMR (DMSO-*d_6_*): 9.3 (br, 3H), 7.7 (d, 2H, *J* = 9 Hz), 7.3 (t, 2H, *J* = 7 Hz), 7.1 (d, 2H, *J* = 9 Hz), 7.0 (d, 2H, *J* = 7 Hz), 6.8 (t, 1H, *J* = 7 Hz), 3.5 (t, 4H, *J* = 5 Hz), 3.4 (t, 2H, *J* = 7 Hz), 3.3 (t, 4H, *J* = 5 Hz), 1.6 (m, 2H, *J* = 7 Hz), 1.3 (m, 10H), 0.9 (t, 3H, *J* = 7 Hz); IR: 3,062, 2,923, 2,853, 1,662, 1,607, 1,505, 1,386, 1,232 cm^-1^; C_25_H_36_N_4_·HCl (428.27). Calc.: C, 69.99; H, 8.69; N, 13.06. Found: C, 69.94; H, 8.68; N, 13.04.

*N-Dodecyl 4-(4-phenylpiperazine-1-yl)benzenecarboximidamide hydrochloride salt* (**18**). Prepared by treatment of the crude imidate **4** with dodecylamine (15 mmol, 3.5 mL) in refluxing ethanol (10 mL) for 1 hour. The precipitate obtained after cooling was filtered and washed with ether and water. Yield: 51%. M.p.: 275–280 °C; ^1^H-NMR (DMSO-*d_6_*): 9.3 (br, 3H), 7.7 (d, 2H, *J* = 9 Hz), 7.3 (t, 2H, *J* = 7 Hz), 7.1 (d, 2H, *J* = 9 Hz), 7.0 (d, 2H, *J* = 7 Hz), 6.8 (t, 1H, *J* = 7 Hz), 3.5 (t, 4H, *J* = 5 Hz), 3.4 (t, 2H, *J* = 7 Hz), 3.3 (t, 4H, *J* = 5 Hz), 1.6 (m, 2H, *J* = 7 Hz), 1.4 (m, 18H), 0.9 (t, 3H, *J* = 7 Hz); IR: 3,047, 2,920, 2,850, 1,668, 1,608, 1,515, 1,466, 1,387, 1,233 cm^-1^; C_29_H_44_N_4_·HCl (484.33). Calc.: C, 71.79; H, 9.35; N, 11.55. Found: C, 71.77; H, 9.34; N, 11.57.

*N-Benzyl 4-(4-phenylpiperazine-1-yl)benzenecarboximidamide hydrochloride salt* (**19**). Prepared by treatment of the crude imidate **4** with benzylamine (15 mmol, 1.6 mL) in refluxing ethanol (10 mL) for 1 hour. After cooling, the precipitate obtained by addition of ether was filtered and washed with water. Yield: 67%. M.p.: 290–295 °C; ^1^H-NMR (DMSO-*d_6_*): 9.3 (br, 3H), 7.7 (d, 2H, *J* = 8 Hz), 7.4 (m, 5H), 7.3 (t, 2H, *J* = 7 Hz), 7.1 (d, 2H, *J* = 8 Hz), 7.0 (d, 2H, *J* = 7 Hz), 6.8 (t, 1H, *J* = 7 Hz), 4.7 (s, 2H), 3.5 (t, 4H, *J* = 5 Hz), 3.3 (t, 4H, *J* = 5 Hz); IR: 3,033, 2,359, 1,665, 1,516, 1,497, 1,452, 1,388, 1,230 cm^-1^; C_24_H_26_N_4_·HCl (406.19). Calc.: C, 70.83; H, 6.69; N, 13.77. Found: C, 70.77; H, 6.66; N, 13.76.

*N-Phenethyl 4-(4-phenylpiperazine-1-yl)benzenecarboximidamide hydrochloride salt* (**20**). Prepared by treatment of the crude imidate **4** with phenethylamine (15 mmol, 1.9 mL) in refluxing ethanol (10 mL) at for 1 hour. After cooling, the precipitate obtained by addition of ether was filtered and washed with water. Yield: 64%. M.p.: >300 °C; ^1^H-NMR (DMSO-*d_6_*): 9.3 (br, 3H), 7.7 (d, 2H, *J* = 8 Hz), 7.4 (m, 5H), 7.3 (t, 2H, *J* = 7 Hz), 7.1 (d, 2H, *J* = 8 Hz), 7.0 (d, 2H, *J* = 7 Hz), 6.8 (t, 1H, *J* = 7 Hz), 3.7 (t, 2H, *J* = 7 Hz), 3.5 (t, 4H, *J* = 5 Hz), 3.3 (t, 4H, *J* = 5 Hz), 3.0 (t, 2H, *J* = 7Hz); IR: 3,051, 1,605, 1,671, 1,498, 1,452, 1,383, 1,231 cm^-1^; C_25_H_28_N_4_·0.8 HCl (420.21). Calc.: C, 72.58; H, 7.02; N, 13.54. Found: C, 72.71; H, 6.89; N, 13.35.

*N-(3-Phenylpropyl) 4-(4-phenylpiperazine-1-yl)benzenecarboximidamide hydrochloride salt* (**21**). Prepared by treatment of the crude imidate **4** with 3-phenylpropylamine (15 mmol, 2.1 mL) in reluxing ethanol (10 mL) for 1 hour. After cooling, the precipitate obtained by addition of ether was filtered and washed with water. Yield: 51%. M.p.: 250–255 °C; ^1^H-NMR (DMSO-*d_6_*): 9.3 (br, 3H), 7.7 (d, 2H, *J* = 8 Hz), 7.4 (m, 5H), 7.3 (t, 2H, *J* = 7 Hz), 7.1 (d, 2H, *J* = 8 Hz), 7.0 (d, 2H, *J* = 7 Hz), 6.8 (t, 1H, *J* = 7 Hz), 3.5 (t, 4H, *J* = 5 Hz), 3.4 (t, 2H, *J* = 7 Hz), 3.3 (t, 4H, *J* = 5 Hz), 2.7 (m, 2H), 1.9 (m, 2H); IR: 3,073, 1,658, 1,607, 1,505, 1,232 cm^-1^; C_26_H_30_N_4_·HCl (434.22). Calc.: C, 71.79; H, 7.18; N, 12.88. Found: C, 71.81; H, 7.2; N, 12.86.

*N-(4-Fluorobenzyl) 4-(4-phenylpiperazine-1-yl)benzenecarboximidamide hydrochloride salt* (**22**). Prepared by treatment of the crude imidate **4** with 4-fluorobenzylamine (15 mmol, 1.7 mL) in refluxing ethanol (10 mL) for 1 hour. After cooling, the precipitate obtained by addition of ether was filtered and washed with water. Yield: 58%. M.p.: 280–285 °C; ^1^H-NMR (DMSO-*d_6_*): 9.3 (br, 3H), 7.8 (d, 2H, *J* = 8 Hz), 7.3 (m, 4H), 7.2 (t, 2H, *J* = 7 Hz), 7.1 (d, 2H, *J* = 8 Hz), 7.0 (d, 2H, *J* = 7 Hz), 6.8 (t, 1H, *J* = 7 Hz), 4.7 (s, 2H), 3.6 (t, 4H, *J* = 5 Hz), 3.3 (t, 4H, *J* = 5 Hz); IR: 3,039, 1,666, 1,600, 1,515, 1,385, 1,234 cm^-1^; C_24_H_25_FN_4_·HCl (424.18). Calc.: C, 67.83; H, 6.17; N, 13.18. Found: C, 67.82; H, 6.18; N, 13.17.

*N-2-(4-Fluorophenyl)ethyl 4-(4-phenylpiperazine-1-yl)benzenecarboximidamide hydrochloride salt* (**23**). The compound was obtained by treatment of the crude imidate **4** with 2-(4-fluorophenyl)-ethylamine (15 mmol, 2.0 mL) in reluxing ethanol (10 mL) for 1 hour. After cooling, a precipitate was obtained by pouring ether into the solution. The solid was filtered and washed with water. Yield: 64%. M.p.: >300 °C; ^1^H-NMR (DMSO-*d_6_*): 9.3 (br, 3H), 7.7 (d, 2H, *J* = 8 Hz), 7.3 (m, 4H), 7.2 (t, 2H, *J* = 7 Hz), 7.1 (d, 2H, *J* = 8 Hz), 7.0 (d, 2H, *J* = 7 Hz), 6.8 (t, 1H, *J* = 7 Hz), 3.7 (t, 2H, *J* = 7 Hz), 3.6 (t, 4H, J = 5 Hz), 3.3 (t, 4H, *J* = 5 Hz), 3.2 (t, 2H, *J* = 7 Hz); IR: 3,040, 1,672, 1,604, 1,511, 1,463, 1,452, 1,385, 1,231, 1,206, 1,160 cm^-1^; C_25_H_27_FN_4_·HCl (438.20). Calc.: C, 68.40; H, 6.43; N, 12.76. Found: C, 68.41; H, 6.39; N, 12.79.

### 3.4. Biological evaluation

To determine the *in vitro* drug susceptibility of *P. carinii*, axenic cultures of the organism were produced as follows. All the experiments were carried out in 24-well plates with a final volume of 2 mL of Dulbecco’s modified Eagle’s medium (DMEM) supplemented with 10% of fetal calf serum containing a final inoculum of 1.0 × 10^6^ organisms per mL. Plates with organisms were incubated for 4 days in an atmosphere of 5% CO_2_ at 37 °C. *P. carinii* was quantitated on air dried smears stained with a rapid panoptic methanol-Giemsa stain (RAL-555), which stains trophic forms, sporocytes and cysts of *Pneumocystis*. All susceptibility assays were set up in triplicate. The anti-*Pneumocystis* activity of a single concentration of compound may be expressed in terms of percent inhibition, defined as the decrease (expressed as percentage) in *P. carinii* forms in antifungal-treated cultures with respect to the total microorganism count in compound-free culture.

## 4. Conclusions 

In summary, a library of 19 monobenzamidines linked on a 4-phenylpiperazine-1-yl scaffold has been synthesized and evaluated *in vitro* against *Pneumocystis carinii*. As in a series of bisbenzamidine analogues, the antifungal activity can easily be modulated by the introduction of appropriate alkyl substituents on the amidine function. However at the lowest concentration (0.1 µg/mL) we evaluated those monobenzamidines, only two derivatives (compounds **7** and **15**) exhibited a percentage of inhibition on the growth of *P. carinii* higher than 50 %. Nevertheless the monobenzamidines reported in this work are obviously less active than pentamidine and other bisbenzamidines already tested. That suggests that the presence of both amidine groups are required to observe a marked anti-*P. carinii* effect.

## Figures and Tables

**Table 1:** Inhibition percentages of the monobenzamidines against *P. carinii*.

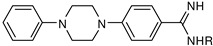				
Compound Number	R	Inhibition % at		
		50.0 µg/mL	10.0 µg/mL	0.1 µg/mL
*Pentamidine* **1**		95.0 ± 2.1	92.0 ± 4.5	76.0 ± 4.9
**5**	H	87.0 ± 4.4	80.0 ± 10.0	23.0 ± 5.0
**6**	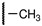	62.0 ± 16.5	56.0 ± 27.6	42.0 ± 12.1
**7**	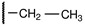	83.0 ± 8.6	87.0 ± 27.2	61.0 ± 15.6
**8**	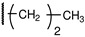	88.0 ± 7.0	72.0 ± 18.3	48.0 ± 31.3
**9**	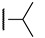	88.0 ± 4.7	90.0 ± 6.9	No activity
**10**	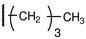	94.0 ± 1.2	92.0 ± 0.6	28.0 ± 14.6
**11**	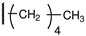	90.0 ± 0.6	94.0 ± 0.3	13.0 ± 16.5
**12**	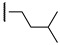	96.0 ± 0.6	91.0 ± 0.6	No activity
**13**	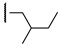	97.0 ± 0.6	92.0 ± 1.1	No activity
**14**	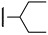	97.0 ± 1.5	93.0 ± 4.9	43.0 ± 3.2
**15**	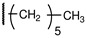	91.0 ± 7.5	96.0 ± 0.6	56.0 ± 2.6
**16**	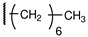	92.0 ± 2.6	90.0 ± 4.0	No activity
**17**	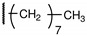	80.0 ± 16.0	93.0 ± 4.3	No activity
**18**	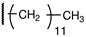	85.0 ± 12.5	79.0 ± 9.7	No activity
**19**	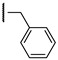	93.0 ± 4.1	91.0 ± 1.4	No activity
**20**	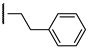	95.0 ± 2.1	94.0 ± 1.15	No activity
**21**	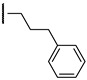	95.0 ± 2.5	96.0 ± 2.3	No activity
**22**	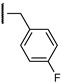	73.0 ± 4.5	70.0 ±9.0	No activity
**23**	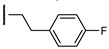	96.0 ± 1.5	69.0 ± 7.6	No activity
**2**		59.0 ± 4.5	66.0 ± 7.5	32.0 ± 7.0
